# Professor W. D. Miller

**Published:** 1894-04

**Authors:** 


### PROFESSOR W. D. MILLER.

Professor Miller has been made a professor in the medical faculty of the University of Berlin. Very few, we imagine, not familiar with German habit of thought and practice, can appreciate what this means. Aside from the fact that he is the first American to fill such a position, he is also the first dentist to reach such a place (German or otherwise). The fact that he had not followed the usual course regarded as indispensable in Germany to make him eligible for the honor renders it, perhaps, one of the most remarkable appointments of modern times. It is also an honor to the University that it is willing to recognize ability, such as Professor Miller possesses, and has demonstrated a power to override national prejudice and long-cherished customs.

